# Effects of Gadolinium-Based Contrast Agents on Thyroid Hormone Receptor Action and Thyroid Hormone-Induced Cerebellar Purkinje Cell Morphogenesis

**DOI:** 10.3389/fendo.2016.00115

**Published:** 2016-08-26

**Authors:** Winda Ariyani, Toshiharu Iwasaki, Wataru Miyazaki, Erdene Khongorzul, Takahito Nakajima, Satomi Kameo, Hiroshi Koyama, Yoshito Tsushima, Noriyuki Koibuchi

**Affiliations:** ^1^Department of Integrative Physiology, Gunma University Graduate School of Medicine, Maebashi, Japan; ^2^Department of Liberal Arts and Human Development, Kanagawa University of Human Services, Kanagawa, Japan; ^3^Department of Diagnostic Radiology and Nuclear Medicine, Gunma University Graduate School of Medicine, Maebashi, Japan; ^4^Department of Public Health, Gunma University Graduate School of Medicine, Maebashi, Japan

**Keywords:** endocrine disruption, cerebellum, neurotoxicology, development, T4, T3

## Abstract

Gadolinium (Gd)-based contrast agents (GBCAs) are used in diagnostic imaging to enhance the quality of magnetic resonance imaging or angiography. After intravenous injection, GBCAs can accumulate in the brain. Thyroid hormones (THs) are critical for the development and functional maintenance of the central nervous system. TH actions in brain are mainly exerted through nuclear TH receptors (TRs). We examined the effects of GBCAs on TR-mediated transcription in CV-1 cells using transient transfection-based reporter assay and TH-mediated cerebellar Purkinje cell morphogenesis in primary culture. We also measured the cellular accumulation and viability of Gd after representative GBCA treatments in cultured CV-1 cells. Both linear (Gd-diethylene triamine pentaacetic acid-bis methyl acid, Gd-DTPA-BMA) and macrocyclic (Gd-tetraazacyclododecane tetraacetic acid, Gd-DOTA) GBCAs were accumulated without inducing cell death in CV-1 cells. By contrast, Gd chloride (GdCl_3_) treatment induced approximately 100 times higher Gd accumulation and significantly reduced the number of cells. Low doses of Gd-DTPA-BMA (10^−8^ to 10^−6^M) augmented TR-mediated transcription, but the transcription was suppressed at higher dose (10^−5^ to 10^−4^M), with decreased β-galactosidase activity indicating cellular toxicity. TR-mediated transcription was not altered by Gd-DOTA or GdCl_3_, but the latter induced a significant reduction in β-galactosidase activity at high doses, indicating cellular toxicity. In cerebellar cultures, the dendrite arborization of Purkinje cells induced by 10^−9^M T_4_ was augmented by low-dose Gd-DTPA-BMA (10^−7^M) but was suppressed by higher dose (10^−5^M). Such augmentation by low-dose Gd-DTPA-BMA was not observed with 10^−9^M T_3_, probably because of the greater dendrite arborization by T_3_; however, the arborization by T_3_ was suppressed by a higher dose of Gd-DTPA-BMA (10^−5^M) as seen in T_4_ treatment. The effect of Gd-DOTA on dendrite arborization was much weaker than that of the other compounds. These results indicate that exposure to specific GBCAs may, at least in part, cause toxic effects in the brain by disrupting the action of THs on TRs. The toxic effects of GBCAs may depend on the chemical structure of GBCA and the dose. Thus, it is very important to choose appropriate GBCAs for imaging to prevent adverse side effects.

## Introduction

Gadolinium (Gd) is a heavy metal of the lanthanide group with an oxidation state of +3 and an ionic radius of 0.99 Å. The trivalent Gd ion (Gd^3+^) exhibits cellular toxicity by inhibiting various Ca^2+^ channels through competitive binding with a much higher affinity than Ca^2+^ (1, 2). In spite of their toxicity, Gd-chelated compounds, such as chelated organic Gd complexes, have been used as contrast agents in magnetic resonance imaging (MRI) and magnetic resonance angiography. Gd-based contrast agents (GBCAs) are chelated forms of Gd that are manufactured to reduce the toxicity of Gd by avoiding the presence of free Gd^3+^ (2). GBCAs are classified as linear or macrocyclic, according to their chelated structure, and as ionic or non-ionic, based on their ion charge (1, 3). In general, compared with linear types, macrocyclic GBCAs are more stable, tend to bind with Gd for longer durations, and have lower dissociation rates (1, 2, 4).

In rodent studies, tissue deposition of Gd by GBCA exposure has been found in the skin, liver, kidney, lung, heart, and bones (5, 6). Once there, the low stability of GBCAs means that they may undergo transmetallation and release free Gd^3+^ by replacement with cations, such as zinc, iron, copper, and calcium. The released Gd^3+^ is then free to attach to endogenous anions, such as phosphate, citrate, hydroxide, and carbonate, and can deposit in tissues as insoluble compounds (4, 7). In some patients with renal insufficiency, exposure to GBCA is known to cause nephrogenic systemic fibrosis (NSF) (8, 9), and although the exact cause remains unclear, GBCA exposure activates toll-like receptor (TLR) 4 and TLR7-mediated gene expression, resulting in increased production of many cytokines, chemokines, and growth factors. These include transforming growth factor beta 1 (TGF-β1) and interleukin-6 (IL-6) in macrophages, and increase production of collagens I and III, fibronectin, and hyaluronic acid in fibroblasts (8–10). As a result of the increased production of these factors, tissue inflammation and fibrosis are activated and may cause NSF.

Gadolinium deposition has also been found in brain tissue, even in patients without severe renal dysfunction (11). Specifically, the dentate nucleus and globus pallidus have shown significantly higher Gd accumulation than other brain regions (2, 12). Moreover, Gd induces cell death by inhibiting mitochondrial function and by inducing oxidative stress, followed by a rapid accumulation of reactive oxygen in primary cultures of rat cortical neurons (13, 14). By contrast, another study showed that administration of GBCAs does not cause any severe neurological alterations, such as seizures (15). On the other hand, long-term oral administration of other lanthanides affects functions of learning and memory, swimming and walking abilities, and touch response behavior in rats (16). Thus, although controversy still exists, lanthanides, including Gd, may cause neurotoxic effects in brain. However, the mechanism that causes the toxic effects of lanthanides may not be fully explained by the disruption of Ca^2+^ channels and mitochondrial function.

The thyroid hormones [THs; 3,5,3′-tri-iodo-l-thyronine (or T_3_) and thyroxine (or T_4_)] play important roles in brain development, including the development of the cerebellum (17, 18). THs regulate various developmental processes from neuronal and glial proliferation to differentiation and neuronal migration in definitive brain regions (19). They also regulate the formation of neuronal cytoarchitecture and synaptogenesis (20). Thus, TH deficiency can change neuronal development and function. In addition, neuronal excitability and neurotransmitter transportation are also affected (21). Consequently, abnormal motor coordination, decreased locomotor activity, and increased anxiety have been observed in hypothyroid patients and experimental animal models (21). It should be noted that some of these phenotypes may also be observed in animals that are exposed to Gd compounds (15, 16).

Thyroid hormone actions are mainly mediated by nuclear TH receptors (TRs) that bind to a specific DNA sequence, called TH response element (TRE), as a homodimer or a heterodimer with the retinoid X receptor (RXR). TRs have two major isoforms, such as TRα and TRβ, which are encoded by separate genes on human chromosomes 17 and 3, respectively. Both TR isoforms are expressed in adult and developing brains (22). The TR–RXR complex recruits a series of cofactors (corepressors and coactivators) in a ligand-dependent manner and regulates the transcription of target genes (22–24). TH target genes include functional proteins that regulate various brain development and maintenance processes as well as mitochondrial functions.

Two important areas of uncertainty exist in the research literature. First, although several heavy metals affect TH activity (25, 26), the effect of Gd on TH-mediated gene expression has not yet been studied. Second, several brain phenotypes caused by Gd exposure are similar to those of hypothyroid animals; however, the mechanism of Gd toxicity in the brain remains unclear. Moreover, the effects of Gd exposure on TR-mediated transcription and TH-mediated brain development have not yet been studied.

Here, we examined the effect of Gd on TR activity and TH-mediated brain development *in vitro*. We employed three different Gd compounds: Gd chloride (GdCl_3_) as a free Gd^3+^ source, Gd-DTPA-BMA (Omniscan™) as a linear Gd, and Gd-DOTA [Dotarem™ (Magnescope™)] as a macrocyclic Gd. We examined the effects of these Gd compounds on cell viability and TR-mediated transcription using CV-1 cells. We also cultured newborn rat cerebellar tissue with Gd compounds to investigate their effect on TH-induced morphological changes in Purkinje cells because this is considered to be a good model for the study of TH action on neuronal morphogenesis and its modification by various chemicals and because the dendrite growth of Purkinje cells provides a reliable index of their function and development (27). Using this model, we found that Gd compounds altered dendritogenesis.

## Materials and Methods

### Chemicals

**Figure 1:**
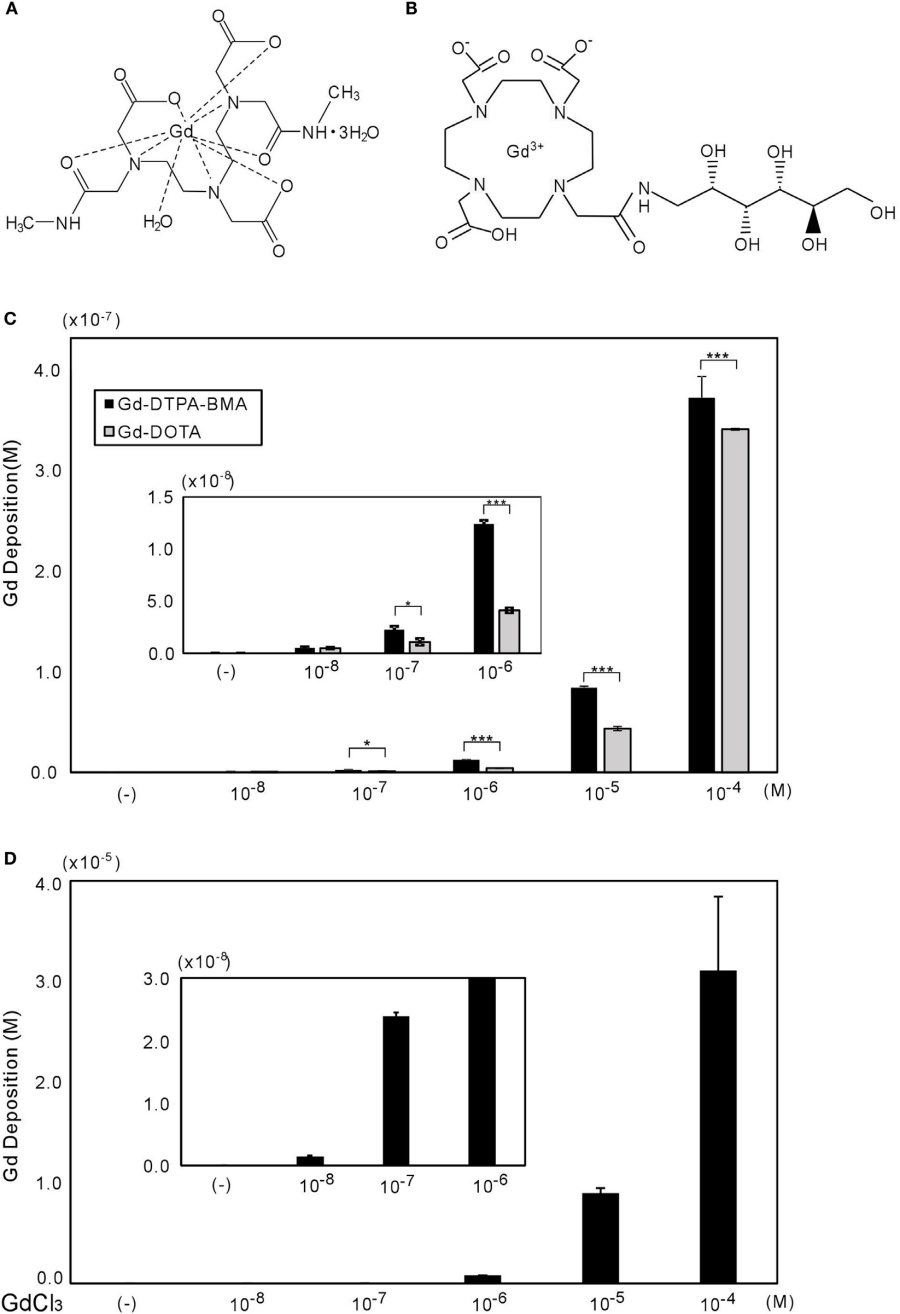
**Chemical structures of Gd-DTPA-BMA (A) and Gd-DOTA (B), with gadolinium deposition shown in CV-1 cells (C,D)**. **(A,B)** molecular structures of Gd-DTPA-BMA and Gd-DOTA, respectively (28). The CV-1 cells were exposed to 10^−8^ to 10^−4^M of Gd-DTPA-BMA, Gd-DOTA, or GdCl_3_ for 24 h. The deposition of total gadolinium was determined by ICP-MS. Cellular Gd deposition (M/well) can be observed after Gd-DTPA-BMA and Gd-DOTA **(C)**, and GdCl_3_ treatment **(D)**. Note that the gadolinium concentration after GdCl_3_ treatment was approximately 100 times higher than that after GBCA treatments. Data are presented as means ± SEM of experiments performed in triplicate. ***p < 0.001 and *p < 0.05 indicate statistical significance by Bonferroni’s test.

Gd chloride (GdCl_3_) hexahydrate (MW 371.70), with 99.9% purity and dissolved in distilled water, was purchased from Sigma (St. Louis, MO, USA) and stored in −20°C until use. Gd-DTPA-BMA (Omniscan™) and Gd-DOTA [Dotarem™ (Magnescope™)] were obtained from their respective manufacturers, and then diluted with distilled water to their final concentrations (Figure 1A). T_3_ (98% purity) (Sigma) was diluted using a vehicle (final concentration of 0.04 NaOH for 10^−3^M of T_3_ stock) as the diluent. T_4_ (98% purity), purchased from Sigma, was diluted with DMSO (for 10^−1^M of T_4_ stock), and then diluted with DMEM/F12 to its final concentration from 10^−1^M.

### Plasmids

Expression vectors for rat TRα1 and TRβ1 have previously been described (29, 30). The luciferase (LUC) reporter constructs, artificial direct repeat TRE, DR4 to TK-LUC (DR4-TRE), and chick lysozyme (F2)-thymidine kinase (TK)-LUC (F2-TRE) have also been described elsewhere (30).

### Clonal Cell Culture

Monkey kidney fibroblast-derived CV-1 clonal cells were maintained in DMEM (Sigma), supplemented with 10% fetal bovine serum deprived of small lipophilic hormone, with 100 U/mL penicillin and 100 μg/mL streptomycin (Invitrogen, San Diego, CA, USA) at 37°C under a 5% CO_2_ atmosphere. The serum was stripped of hormones by constant mixing with 5% (w/v) AGX1-8 resin (Bio-Rad, Hercules, CA, USA) and powdered charcoal before ultrafiltration (31).

### Analysis of Gd Deposition by Inductively Coupled Plasma-Mass Spectrometry

Cells were plated at a density of 1 × 10^5^ cells/mL in six-well plates until 80% confluency was achieved and were incubated in the presence of Gd-DTPA-BMA, Gd-DOTA, or GdCl_3_ for 24 h. After exposure, the cells were washed with phosphate buffered saline (PBS), and 500 μL of 0.25 w/v% Trypsin–1 mmol/L EDTA 4 Na solution (Wako Pure Chemical Industries, Ltd., Tokyo, Japan) was added before they were scraped, centrifuged at 3000 rpm for 3 min, and washed again with PBS three times. The cell lysate was stored at −80°C until used for inductively coupled plasma-mass spectrometer (ICP-MS) measurements.

Each homogenized sample was weighed and sealed in a perfluoroalkoxy vial along with 0.5 mL of nitric acid and 0.1 mL of hydrogen peroxide (Ultrapure reagent, Kanto Chemical, Tokyo, Japan) for hazardous metal analysis, and it was then subjected to sample digestion with eight sequences of a microwave program for 125 min with the MLS 1200 Mega (Milestone Inc., Shelton, CT, USA), which is a high performance microwave digestion unit. After cooling, it was diluted to 10 mL with ultrapure water. The total concentration of Gd^3+^ (free + bound) in the cells was measured with ICP-MS (ELAN DRC II^®^ mass spectrometer, Perkin Elmer Life and Analytical Sciences Inc., Waltham, MA, USA) with the following parameters: ICP RF (radiofrequency) power, 1500 W; plasma gas flow, 17.0 L/min; auxiliary gas flow, 1.20 L/min; and nebulizer gas flow, 0.86 L/min. A standard curve of inorganic Gd^3+^ (0–300 ppm) in 3% HNO_3_ and digested sample solution was monitored by the responses of the stable isotopes ^158^Gd and ^160^Gd. The cellular Gd concentration was determined by multiplying the weight of Gd per milliliter in the digested solution by the dilution factor and dividing it by the weight of the cellular sample.

### Cell Viability Assay

We performed the 3-[4,5-dimethylthiazol-2-yl]-5-[3-carboxymethoxyphenyl]-2-[4-sulfophenyl]-2Htetrazolium (MTS) cell proliferation assay, as described in the instruction manual of the Promega Technical Bulletin CellTiter 96^®^ AQueous One Solution Cell Proliferation Assay. The conversion of MTS into aqueous soluble formazan was caused by the succinic dehydrogenase found in the metabolically active mitochondria. Cells were plated at a density of 1 × 10^5^ cells/0.1 mL in 96-well plates until 80% confluency and were then incubated in the presence of Gd-DTPA-BMA, Gd-DOTA, or GdCl_3_, each for 24, 48, and 96 h, respectively. After exposure to these compounds, the MTS reagent was added to the medium. The absorbance, representing mitochondrial metabolic activity, was measured at 490 nm using a micro-plate reader (Bio-Rad). Relative values were calculated as percentages of the values obtained from the normal control group. All MTS studies were repeated at least three times in triplicate. Data are presented as means ± SEM of one representative experiment performed in triplicate.

### Transient Transfection-Based Reporter Gene Assay

Cells were plated at a density of 1 × 10^5^ cells/mL in 24-well plates until 80% confluency, and then transfected using a calcium–phosphate precipitation method (31). Expression vectors encoding TR (0.02 μg) were cotransfected with reporter (F2-TRE-LUC) (0.2 μg) into CV-1 cells. *Cytomegalovirus*-β-galactosidase plasmid (0.04 μg) was cotransfected as the internal control. At 16–18 h after transfection, cells were incubated with stripped medium, containing the indicated concentration of a vehicle (final concentration of 0.04M NaOH) or ligand (10^−7^M T_3_), and Gd-DTPA-BMA, Gd-DOTA, or GdCl_3_ for 24 h. Cells were then harvested to measure the luciferase activity, as described elsewhere (31). Total amounts of DNA (0.26 μg) per well were balanced by adding pcDNA3 plasmids (Invitrogen). LUC activities were normalized to β-galactosidase activity and then calculated as relative LUC activities. All transfection studies were repeated at least three times in triplicate. Data shown represent mean ± SEM of one representative experiment performed in triplicate.

### Primary Cerebellar Culture

A pregnant Wistar rat was purchased from Japan SLC (Hamamatsu, Japan), and newborn rats were euthanized under isoflurane anesthesia on the first day of birth. The animal experiment protocol in the present study was approved by the Animal Care and Experimentation Committee, Gunma University, and all efforts were made to minimize the number of animals used and their suffering. Details of the culture methods have been previously described (32). Briefly, cerebellar tissue was digested with 0.2 U/mL of papain (Worthington, Lakewood, NJ, USA) in PBS containing 0.2 mg/mL l-cysteine, 0.2 mg/mL bovine serum albumin (Intergen Company, Purchase, NY, USA), 5 mg/mL glucose, and 0.02 mg/mL DNase I (Sigma, 400–600 U/mg). This was continued for 25 min with continued shaking at 36.5°C. Dissociated cells were suspended in serum-free medium without THs and plated at a density of 3 × 10^5^ cells/0.3 mL in the wells of chamber slides (Lab-Tek 8-mm-diameter wells, Nalge Nunc International, Rochester, NY, USA) pre-coated with 0.1 mg/mL poly-l-lysine (Sigma). Then, at 16–24 h after plating, T_4_ (10^−9^M) or T_3_ (10^−9^M), and/or Gd-DTPA-BMA, Gd-DOTA, and GdCl_3_ were added to the culture medium. One half of the medium was replaced with fresh medium every 3 days, and the mixed cerebellar cells were cultured in a 5% CO_2_ incubator at 37°C for 17 days. The effect of dimethyl sulfoxide was excluded using control and experimental media at a final concentration of 0.01%, and by avoiding freezing and thawing.

### Immunocytochemistry for Calbindin to Analyze Purkinje Cell Development

Immunocytochemistry of the cultured cells was performed, as previously described (32). Briefly, the Purkinje cells underwent immunochemical staining with a mouse monoclonal anti-calbindin-28K antibody (1:200 Sigma) and a donkey anti-mouse IgG (H + L) secondary antibody, Alexa Fluor^®^ 488 conjugate (1:200; Thermo Fisher Scientific Inc., Waltham, MA, USA), and were then inspected under a laser confocal scanning microscope (FV1000D spectral type inverted Microscope IX81, Olympus, Tokyo, Japan, and ZEISS LSM 880, Carl Zeiss Microscopy GmbH, Jena, Germany). In some cultures, the cell nuclei were also stained with 4′,6-diamidino-2-phenylindole (DAPI). To quantify dendrite arborization, the total area covered by the dendritic tree on 15 randomly selected Purkinje cells per experiment was determined by tracing the outline of the cell and its dendritic branches, before computing the total area using ImageJ software (NIH). The numbers of Purkinje cells (calbindin-positive cells) and DAPI-positive nuclei per well (1 cm^2^) were also counted. Data are presented as means ± SEM, and the typical result from one experiment is shown graphically. More than three independent experiments were performed, and the results were consistent for each experiment.

### Statistical Analysis

Treatment-related effects were analyzed using analysis of variance. *Post hoc* comparison was made using Bonferroni’s test. All *p*-values <0.05 were considered to be statistically significant.

## Results

### Gadolinium Deposition in CV-1 Cells

Gadolinium concentration in CV-1 cells after 24 h of exposure was measured by ICP-MS (Figure 1B). Gd-DTPA-BMA exposure (10^−8^ to 10^−4^M) induced Gd accumulation in CV-1 cells, ranging from 3 × 10^−10^ to 4 × 10^−7^M. Although Gd-DOTA also induced Gd accumulation at the same dose ranges, the level of Gd deposition was significantly lower than that induced by Gd-DTPA-BMA. As a positive control, we also measured Gd deposition after GdCl_3_ treatment. In solution, Gd^3+^ freely dissociates from Cl^−^, and consistent with this, the levels of Gd were approximately 100 times greater than those after GBCA treatment (Figures 1C,D). However, it should be noted that we observed many precipitates in the culture medium when the GdCl_3_ concentration exceeded 10^−7^M. This is consistent with previous studies showing that Gd may be precipitated as Gd phosphate or may promote precipitation of calcium–phosphate (10, 33).

### Effects of GBCA Exposure on Cell Viability

**Figure 2:**
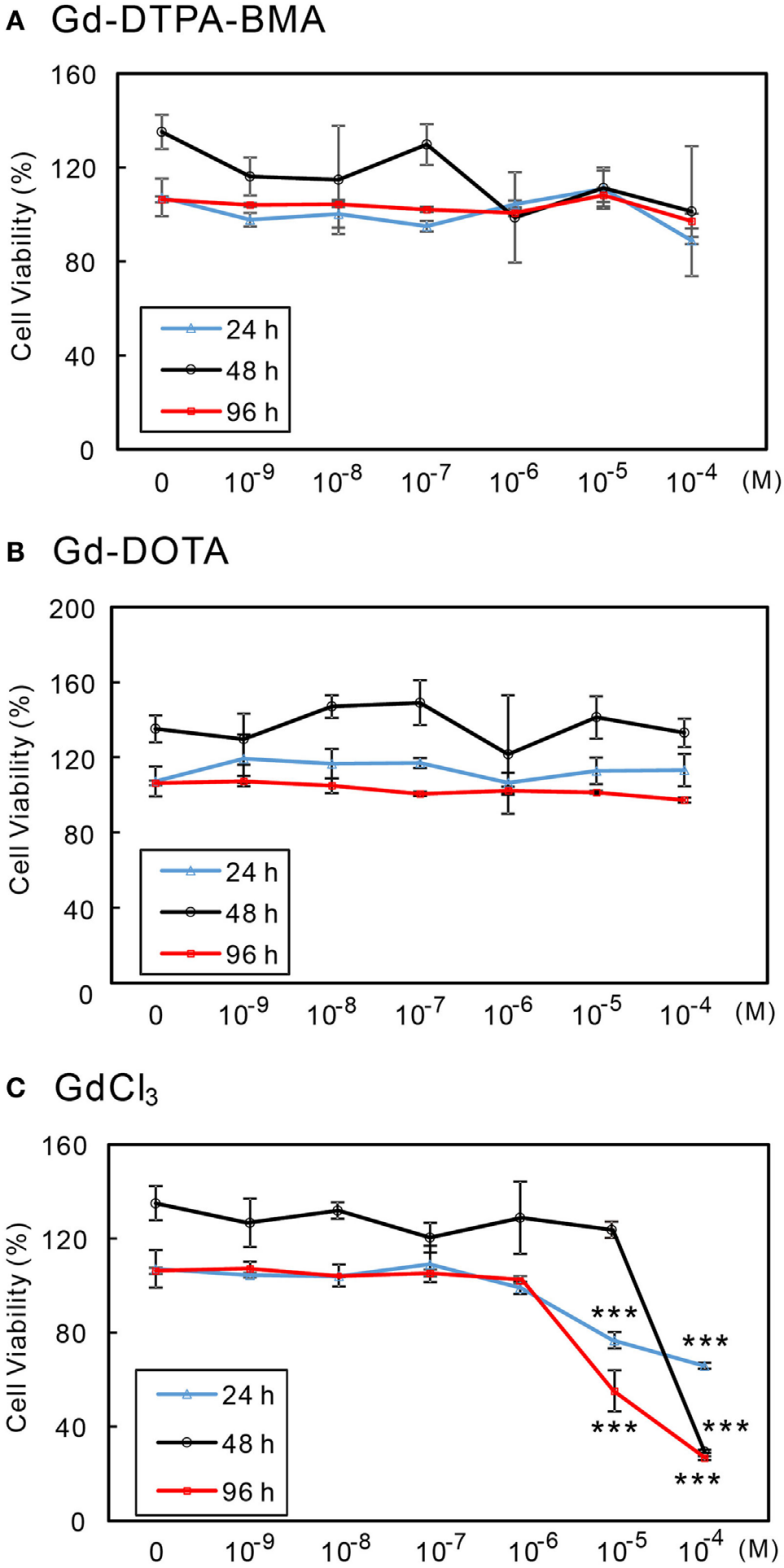
**Effects of GBCA or GdCl_3_ exposure on the cellular viability**. CV-1 cells were exposed to several concentrations of Gd-DTPA-BMA **(A)**, Gd-DOTA **(B)**, and GdCl_3_ **(C)**, each for 24, 48, and 96 h, respectively. Cell viability was determined by MTS assay and calculated as a percentage of the control viability. Data are presented as means ± SEM of experiments performed in triplicate. ***p < 0.001 indicates statistical significance by Bonferroni’s test compared with the negative control (no Gd treatment).

The effects of Gd-DTPA-BMA, Gd-DOTA, and GdCl_3_ exposure on the viability of CV-1 cells were examined by MTS cell proliferation assay. Gd-DTPA-BMA and Gd-DOTA exposure did not affect cell viability (Figures 2A,B). By contrast, GdCl_3_ reduced cell viability by 60% at 24 h, 29% for 48 h, and 20% at 96 h at a level of 10^−4^M (Figure 2C). These results indicate that GBCA exposure does not induce CV-1 cell death.

### Alteration of TR-Mediated Transcription Induced by Gd-DTPA-BMA

**Figure 3:**
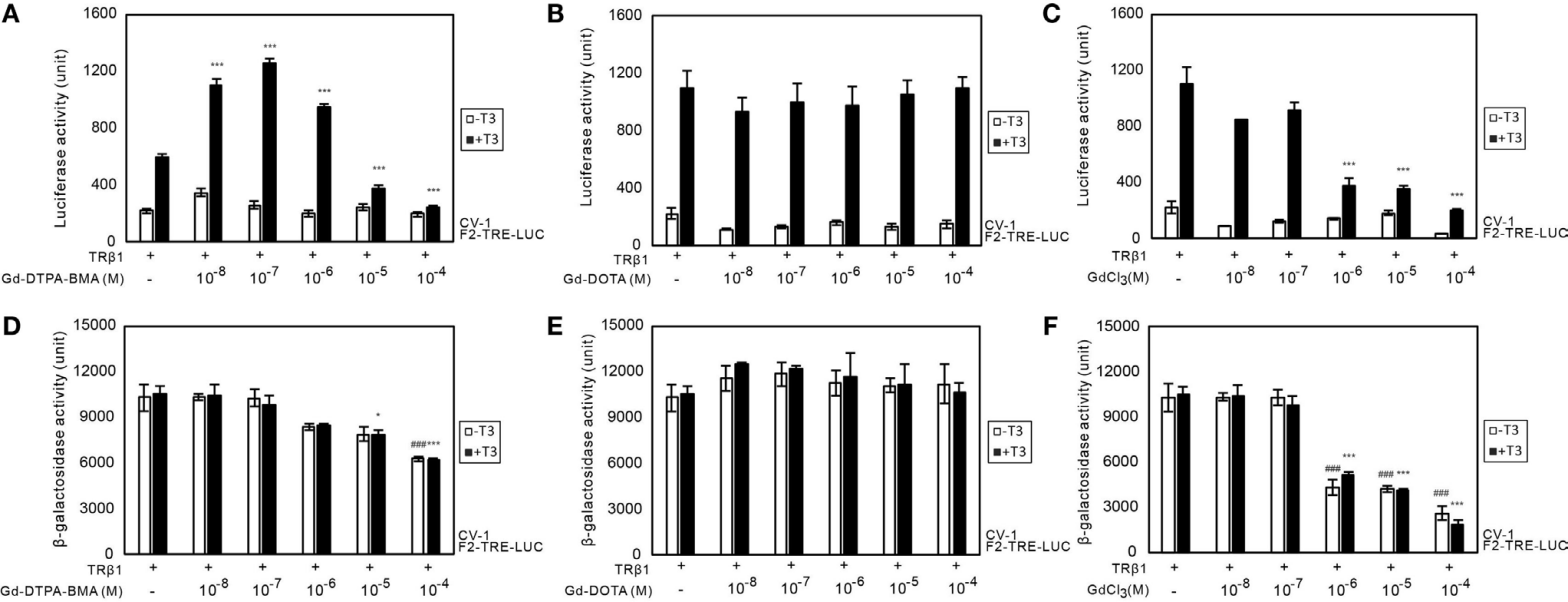
**Effects of GBCA or GdCl_3_ on TR-mediated transcription**. Expression plasmids encoding TRβ1 were cotransfected with F2-TK-LUC into CV-1 cells. Cells were cultured with or without 10^−7^ T_3_ and the indicated amount of Gd-DTPA-BMA **(A,D)**, Gd-DOTA **(B,E)**, or GdCl_3_ **(C,F)**. To compare the general cellular transcriptional activity, luciferase and β-galactosidase activities in the cell lysate after Gd-DTPA-BMA **(A,D)**, Gd-DOTA **(B,E)**, or GdCl_3_ **(C,F)** treatment are shown. The total amount of DNA per well was balanced by adding a pcDNA3 vector. Data are presented as means ± SEM of experiments performed in triplicate. ***p < 0.001, **p < 0.01, and *p < 0.05 indicate statistical significance by Bonferroni’s test compared with TRβ1 (+), T_3_ (+), and Gd-DTPA-BMA, Gd-DOTA, or GdCl_3_ (−). ^###^*p* < 0.001 indicates statistical significance by Bonferroni’s test compared with TRβ1 (+), T_3_ (−), and Gd-DTPA-BMA, Gd-DOTA, or GdCl_3_ (−).

We performed transient transfection-based reporter gene assay in CV-1 cells to investigate the effect of Gd-DTPA-BMA, Gd-DOTA, and GdCl_3_ on TR-mediated transcription (Figure 3). In the presence of T_3_ (10^−7^M), lower doses of Gd-DTPA-BMA (10^−8^ to 10^−6^M) augmented TRβ1-mediated transcription through F2-TRE, whereas transcription was suppressed by higher doses of Gd-DTPA-BMA (10^−5^ to 10^−4^M) (Figure 3A). However, exposure to Gd-DOTA did not change the TRβ1-mediated transcription through F2-TRE in the presence of T_3_ at the same level (10^−7^M) (Figure 3B). Suppression of transcription was also caused by high-dose GdCl_3_ (Figure 3C). We also performed similar analyses using TRα instead of TRβ and using DR4-TRE instead of F2-TRE. The effect was essentially the same, even when we changed the TR–TRE combination (data not shown). When we carefully examined the changes in β-galactosidase activities (Figures 3D–F) to evaluate the change in cellular function, we found that after Gd-DTPA-BMA treatment, β-galactosidase activity tended to decline with increase in its concentration (Figure 3D). Thus, the decline in transcription that was observed with higher doses of Gd-DTPA-BMA may not be due to direct inhibition of TR-mediated transcription but rather by inhibition of cellular function. During treatment with GdCl_3_, β-galactosidase activity was significantly decreased as the concentration increased, indicating that Gd^3+^ disrupted cellular function (Figure 3F). In the groups treated with Gd-DOTA, β-galactosidase activity was not altered (Figure 3E), regardless of the dose, indicating that Gd-DOTA did not affect TR-mediated transcription.

### Exposure to Gd Compounds Impaired the TH-Dependent Dendrite Arborization of Purkinje Cells and Induced Purkinje Cell Death in Primary Cerebellar Culture

**Figure 4:**
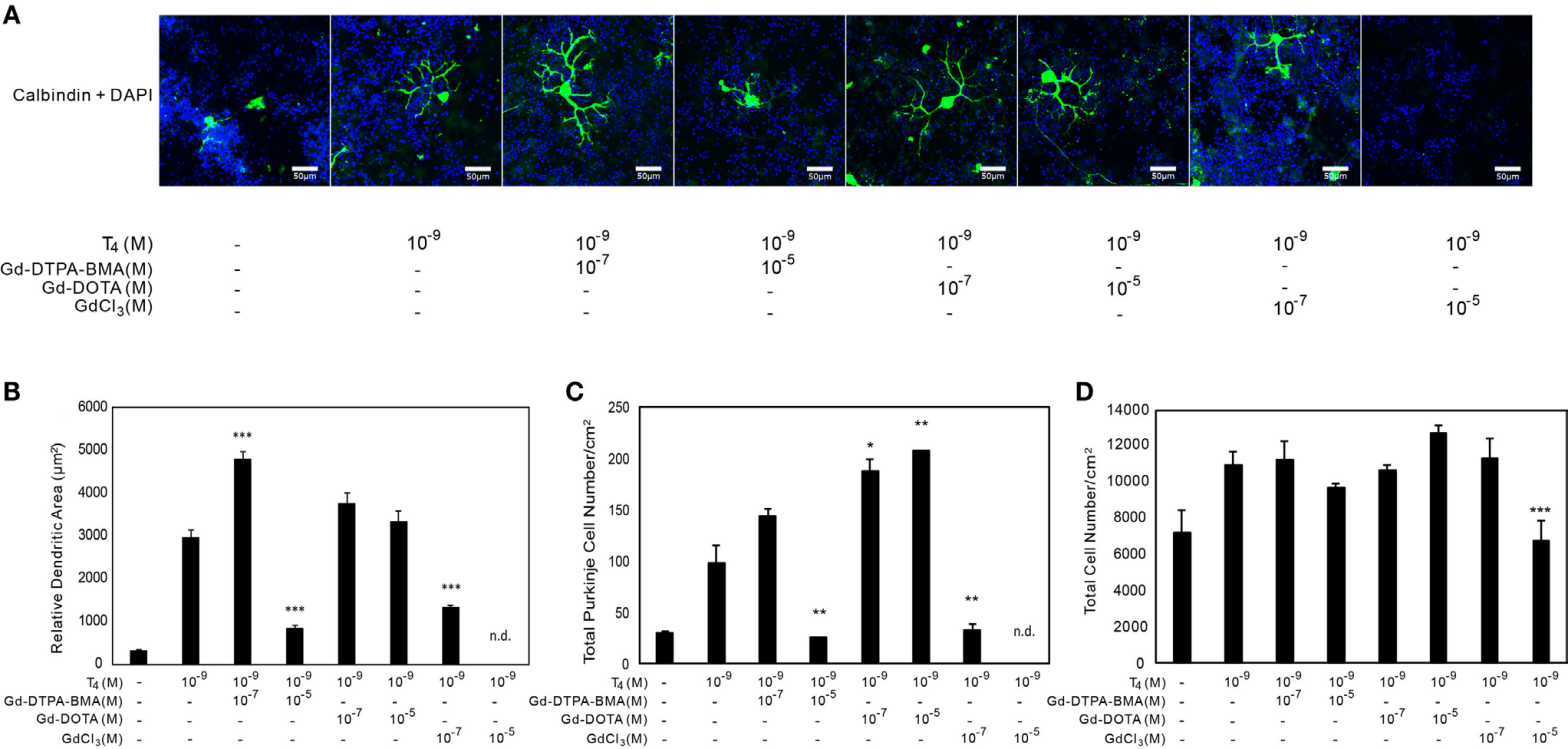
**Effects of GBCA or GdCl_3_ on the T_4_-induced arborization of Purkinje cell dendrites and the relative number of Purkinje cells**. Cerebellar cells were cultured for 17 days followed by immunohistochemical analysis with calbindin and DAPI. **(A)** Representative photomicrographs showing the effects of Gd-DTPA-BMA, Gd-DOTA, or GdCl_3_ on Purkinje cell morphology. Bars indicate 50 μm. **(B)** Change in the dendritic areas of Purkinje cells following Gd-DTPA-BMA, Gd-DOTA, or GdCl_3_ treatment. **(C)** Change in the number of Purkinje cells/well (1 cm^2^) following Gd-DTPA-BMA, Gd-DOTA, or GdCl_3_ treatment. **(D)** Total number of DAPI-positive nuclei/well (1 cm^2^) following Gd-DTPA-BMA, Gd-DOTA, or GdCl_3_ treatment. Dendritic areas and total number of DAPI-positive nuclei were quantified using ImageJ software (NIH), and the total number of Purkinje and total cells/well was manually counted. Data are expressed as means ± SEM (*n* = 15 determinations) and are representative of at least three independent experiments. ***p < 0.001, **p < 0.01, and *p < 0.05 indicate statistical significance by Bonferroni’s test compared with T_4_ (+), and Gd-DTPA-BMA, Gd-DOTA, or GdCl_3_ (−).

**Figure 5:**
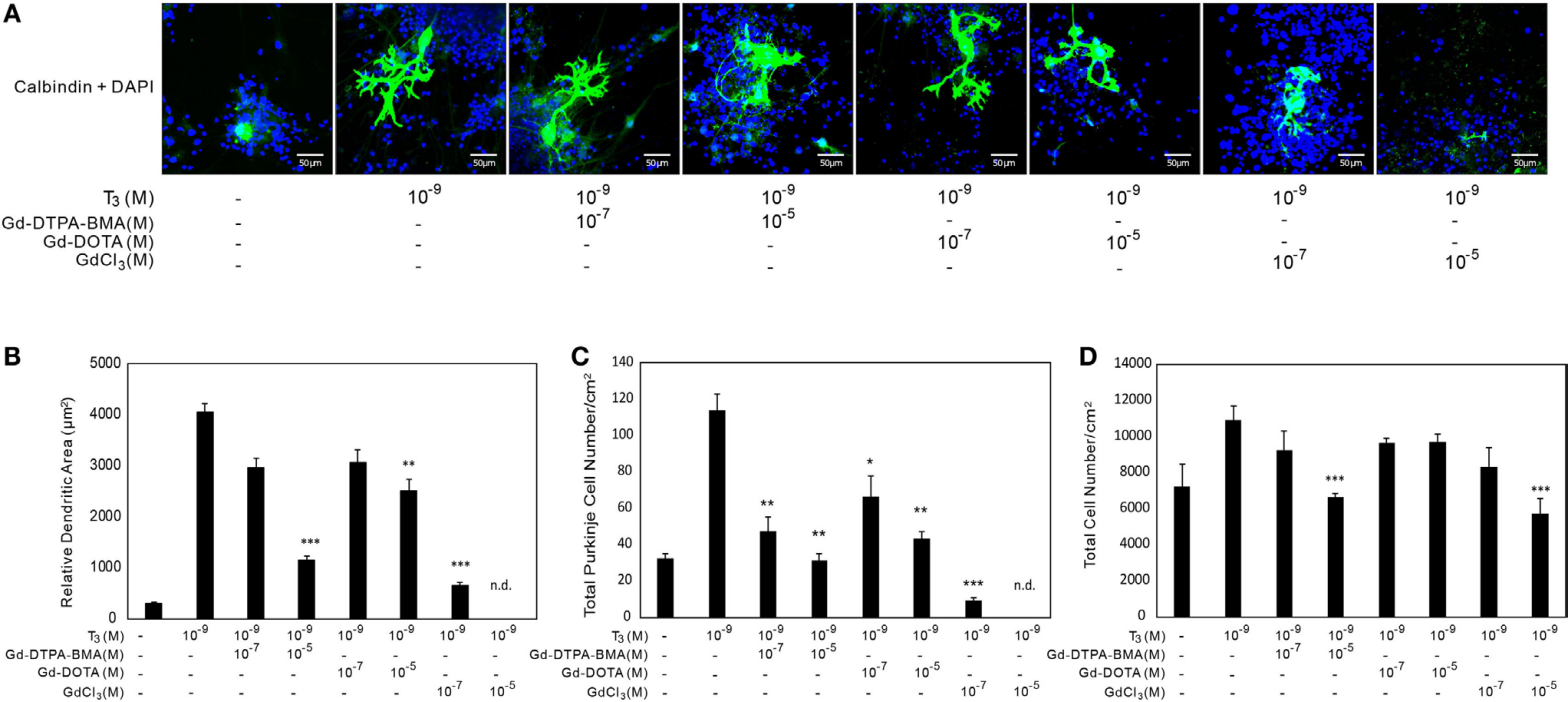
**Effects of GBCA or GdCl_3_ on the T_3_-induced arborization of Purkinje cell dendrites and the relative number of Purkinje cells**. Cerebellar cells were cultured for 17 days followed by immunocytochemical analysis with calbindin and DAPI. **(A)** Representative photomicrographs showing the effects of Gd-DTPA-BMA, Gd-DOTA, or GdCl_3_ on Purkinje cell morphology. Bars indicate 50 μm. **(B)** Change in the dendritic areas of Purkinje cells following Gd-DTPA-BMA, Gd-DOTA, or GdCl_3_ treatment. **(C)** Change in the number of Purkinje cells/well (1 cm^2^) following Gd-DTPA-BMA, Gd-DOTA, or GdCl_3_ treatment. **(D)** Total number of DAPI-positive nuclei/well (1 cm^2^) following Gd-DTPA-BMA, Gd-DOTA, or GdCl_3_ treatment. Dendritic areas and total number of DAPI-positive nuclei were quantified using ImageJ software (NIH), and the total number of Purkinje and total cells/well was manually counted. Data are expressed as means ± SEM (*n* = 15 determinations) and are representative of at least three independent experiments. ***p < 0.001, **p < 0.01, and *p < 0.05 indicate statistical significance by Bonferroni’s test compared with T_4_ (+), and Gd-DTPA-BMA, Gd-DOTA, or GdCl_3_ (−).

The results using CV-1 cells showed that Gd may partly disrupt TH action and cause adverse effects. Consequently, we examined the effect of Gd-DTPA-BMA, Gd-DOTA, and GdCl_3_ exposure on TH-mediated Purkinje cell development in a culture of newborn rat cerebellar tissue. The cerebellar cells of newborn pups were cultured with TH and/or Gd compounds for 17 days and then stained with anti-calbindin-28K antibody and DAPI (Figures 4 and 5). In the T_4_-treated cultures, the cells were distributed evenly in the plate (Figure 4), whereas in T_3_-treated cultures, the cells tended to aggregate in the plate (Figure 5). These results are consistent with previous study (34). Purkinje cells were located within such cellular aggregates in T_3_-treated cultures. T_4_ or T_3_ treatment markedly promoted the dendrite arborization of Purkinje cells. Exposure to 10^−7^M Gd-DTPA-BMA significantly augmented the 10^−9^M T_4_-induced arborization, whereas higher doses of Gd-DTPA-BMA (10^−5^M) dramatically inhibited it (Figure 4A). By contrast, when 10^−9^M T_3_ was used, Gd-DTPA-BMA (10^−7^M) did not augment the dendrite arborization (Figure 5A), probably because T_3_-induced dendrite arborization was much greater than for T_4_. On the other hand, 10^−5^M Gd-DTPA-BMA significantly inhibited the T_3_-induced dendrite arborization. Gd-DOTA did not affect T_4_-induced dendrite arborization (Figure 4A), whereas T_3_-induced dendrite arborization was weakly suppressed by high doses of Gd-DOTA (Figure 5A). GdCl_3_ with T_4_ or T_3_ treatment markedly suppressed the arborization, even at a dose of 10^−7^M. In T_4_-treated cultures, the number of Purkinje cells decreased with high doses of Gd-DTPA-BMA or any doses of GdCl_3_ (Figure 4C). In T_3_-treated cultures, on the other hand, treatment of all Gd compounds decreased the number of Purkinje cells (Figure 5C). The potency to decrease the number was GdCl_3_ > Gd-DTPA-BMA > Gd-DOTA. Furthermore, in T_4_-treated cultures, 10^−5^M GdCl_3_ reduced the total number of cells (Figure 4D), whereas in T_3_-treated cultures, 10^−5^M Gd-DTPA-BMA or GdCl_3_ treatment reduced the total number of cells (Figure 5D).

## Discussion

Here, we initially hypothesized that GBCAs may accumulate in cells and cause adverse effects by disrupting TH action in the brain. Consequently, we studied the effects of GBCA on cellular Gd concentration, TR-mediated transcription, and TH-mediated arborization of cultured Purkinje cell dendrites. We showed that Gd accumulated in CV-1 cells after GBCA and GdCl_3_ treatment for 24 h. Although TR-mediated transcription and T_4_-mediated Purkinje cell dendrite arborization were augmented by low-dose Gd-DTPA-BMA but suppressed by high-dose of Gd-DTPA-BMA, Gd-DOTA at comparable levels did not alter T_4_-mediated dendrite arborization. On the other hand, T_3_ induced greater arborization than T_4_. Under such circumstances, the low-dose Gd-DTPA-BMA-induced augmentation seen with T_4_ was not detected, whereas high doses of Gd-DTPA-BMA suppressed T_3_-mediated Purkinje cell dendrite arborization. On the other hand, GdCl_3_ suppressed cellular function, as evidenced by suppressed β-galactosidase activity and its marked suppression of TH-mediated dendrite arborization in primary cultures. Furthermore, high-dose Gd-DTPA-BMA in T_4_-treated cultures and all GBCA compounds in T_3_-treated cultures significantly reduced the number of Purkinje cells. These results indicate that not only the Gd ion but also chelated Gd compounds may adversely affect the brain by disrupting TH action.

Another interesting finding of the present study is the difference of GBCA action in T_4_- and T_3_-treated cultures. As stated above, the cellular distribution in cultures differed between these two treatments in that cells were more aggregated in the T_3_-treated cultures. A previous study has indicated that the reduced cellular migration in T_3_-treated cultures is due to disruption of integrin α3β1 and extracellular matrix interaction, which is activated by T_4_ or reverse T_3_, but not by T_3_ (34). Purkinje cells were usually found in the regions where the cells were concentrated. Dendrite arborization was augmented by both TH treatments. T_3_ showed a greater effect (1.33×) and this greater amount of arborization may have masked low-dose Gd-DTPA-BMA-induced augmentation of arborization seen in T_4_-treated cultures. On the other hand, while low-dose Gd-DTPA-BMA or any dose of Gd-DOTA did not alter the number of Purkinje cell in T_4_-treated cultures (Figure 4C), all Gd compounds reduced the number of Purkinje cells in the T_3_-treated cultures (Figure 5C). The effect of Gd compounds on the total number of cells was also greater in the T_3_-treated cultures. Although the exact reason for this difference remains unclear, a reduction of cell-to-cell interaction in T_3_-treated cultures may have enhanced the toxicity of the Gd compounds.

Here, although cellular Gd accumulation was much lower with GBCA treatment than with GdCl_3_ treatment, it was unclear whether GBCA itself or dissociated Gd^3+^ was deposited in the cells. A previous study demonstrated that although de-chelation and release of dissociated Gd^3+^ are observed in Gd-DTPA-BMA, the Gd-DOTA form is stable (35). This is consistent with our results, showing higher Gd accumulation with Gd-DTPA-BMA treatment than with Gd-DOTA treatment. Thus, we assume that most of the Gd deposition in this study was caused by dissociated Gd^3+^.

After dissociation, Gd^3+^ may bind to anions (phosphate, carbonate, and hydroxide) in the body fluid to form insoluble salts, which may be deposited in cells where they may cause adverse effects (33). The results of our study support this hypothesis because the level of toxicity (cell viability, β-galactosidase activity, and decrease in dendrite arborization) correlated with the magnitude of dissociation (GdCl_3_ > Gd-DTPA-BMA > Gd-DOTA). However, at a concentration of 10^−4^M, although Gd-DTPA-BMA and Gd-DOTA showed similar Gd accumulation, only Gd-DTPA-BMA suppressed TR-mediated transcription, decreased β-galactosidase expression, and decreased dendrite arborization. Additional mechanisms may be involved in causing such differences.

It is necessary to resolve whether dissociated Gd^3+^ or GBCA enters the cell. As mentioned earlier, dissociated Gd^3+^ may bind to anions, such as phosphate, which can then precipitate, bind with the plasma membrane, and disrupt cellular function, such as blocking calcium channels. We could not find any previous study showing that extracellular Gd- or Gd-chelated compounds enter cells by endocytosis, channels, or transporters. Here, however, low-dose Gd-DTPA-BMA was shown to augment TR-mediated transcription without altering β-galactosidase activity, as well as T_4_-activated Purkinje cell dendritogenesis. Inhibition of the activity of nuclear TR suppresses dendritogenesis in Purkinje cells, and this process is mediated by THs (36). These results indicate that Gd-DTPA-BMA suppressed TH activity in the nuclei. Although the mechanism causing such a disruption is unclear, several possibilities can be considered.

The first possibility is that disruption of calcium signaling by Gd-DTPA-BMA may alter the action of TRs. TH-mediated transcription is partly regulated by Ca^2+^/calmodulin-dependent protein kinase type IV (CaMKIV) (37), whose activity is triggered by Ca^2+^/calmodulin binding. Disruption of intracellular Ca^2+^ homeostasis by blocking calcium channels with Gd-DTPA-BMA may alter CaMKIV activity, leading to alteration of TR-mediated transcription. Another possibility is the disruption of membrane receptor-mediated TH action. In addition to nuclear TR, THs, particularly T_4_, also bind to integrin αvβ3 (38), which mediate various intracellular events through the protein kinase C/phospholipase C pathway. Previous studies indicate that this protein mediates the shuttling of both TRβ and TRα from the cytoplasm to the nucleus (39, 40). However, Gd-DTPA can be used for integrin αvβ3 imaging (41). Integrin αvβ3 is expressed in CV-1 cells (42) and astrocytes (43), where it mediates TH action in Purkinje cells by the conversion of T_4_ to T_3_. Thus, our results are consistent with the hypothesis that Gd-DTPA-BMA may bind to integrin αvβ3 and modulate TH action. However, it should be noted that although Gd-DTPA-BMA treatment showed a biphasic effect on TH activity (augmentation at low doses and suppression at high doses), the mechanism behind this probably depends on the dose, with the disruption of TR-mediated transcription at low doses and suppression of cellular function at higher doses. However, further experiments are required to clarify whether the disruption of TH-mediated action by Gd compounds are caused by disrupting the integrin αvβ3 pathway in astrocytes or by disrupting TR action in Purkinje cells, or both.

Gd-based contrast agents are deposited in the skin, liver, kidney, lung, heart, spleen, diaphragm, and femoral muscle of rats (5, 6). Skin accumulation of GBCAs may cause NSF, particularly in patients with renal insufficiency (9, 44). This is relevant to this study because Gd deposition is also observed in the brain (11), and severe behavioral changes resulted from the administration of GBCA to rat brains (15). Administration of other lanthanides has also been associated with impaired learning and memory (16). Here, we also observed suppression of TH-induced dendritogenesis in the Purkinje cells. Together, these results indicate that the abnormal behavioral alteration following Gd administration may result, at least in part, from the disruption of TH activity in the brain; however, further study is needed to confirm the mechanism. Ishitobi et al. reported that perinatal exposure to Cd suppressed the mRNA expression of neurogranin (RC3), which is a TH-regulated gene that may play an important role in memory and learning (45). It is possible that such alteration of TH-mediated gene expression may cause adverse effects.

Chelated Gd in linear or macrocyclic forms was originally developed to reduce the toxic effects of free Gd. GBCAs are used as clinically approved MRI contrast agents, and a typical ligand used to create a MRI contrast agent has eight donor atoms (1). As discussed earlier, chelated Gd has different stabilities, with ionic-macrocyclic Gd-DOTA being the most stable and non-ionic linear chelates, i.e., gadodiamide (Gd-DTPA-BMA) and gadoversetamide, being the least stable (7). These different characteristics may be the reason for the different effects between Gd-DOTA and Gd-DTPA-BMA in the brain. Thus, appropriate use of GBCA is crucial to avoid adverse effects in the brain. Gd compounds have been found to be accumulated in biological and environmental samples, including surface waters near populated areas all over the world. GBCA application during MRI has been shown to elevate accumulation of Gd in the water surface (46). This is important because we showed that low-dose Gd-DTPA-BMA could alter TR-mediated transcription. Thus, more attention should be paid to environmental Gd pollution.

Therefore, GBCAs were deposited in CV-1 cells, but only Gd-DTPA-BMA altered TR-mediated transcription, with possible augmentation of TR action at low doses and inhibition of cellular function at high doses. Arborization of Purkinje cell dendrites was also altered by GBCAs in both T_3_- and T_4_-treated cultures. Gd-DOTA showed weak suppression of dendrite arborization at high doses and decrease in the number of Purkinje cells only in T_3_-treated cultures, in which cell-to-cell interaction may not be as stable as those in T_4_-treated cultures. Thus, this compound was considered to be safer compared with Gd-DTPA-BMA. These results indicate that an appropriate GBCA should be used to avoid adverse effects. In addition, the possibility that Gd may act as an endocrine-disrupting chemical at low doses requires further attention.

## Author Contributions

WA, TI, and WM conducted the complete experiment and prepared the data and manuscript. YT and NK had responsibility for the whole experiment, earned the grant, made the strategy, and prepared the manuscript. EK and TN contributed by experimental support. SK and HK were responsible for the ICP-MS measurements.

## Conflict of Interest Statement

The authors declare that the research was conducted in the absence of any commercial or financial relationships that could be construed as a potential conflict of interest.
